# Successful recruitment to trials: findings from the SCIMITAR+ Trial

**DOI:** 10.1186/s13063-018-2460-7

**Published:** 2018-01-19

**Authors:** Emily Peckham, Catherine Arundel, Della Bailey, Tracy Callen, Christina Cusack, Suzanne Crosland, Penny Foster, Hannah Herlihy, James Hope, Suzy Ker, Tayla McCloud, Crystal-Bella Romain-Hooper, Alison Stribling, Peter Phiri, Ellen Tait, Simon Gilbody, on behalf of the SCIMITAR+ collaborative

**Affiliations:** 1 Department of Health Sciences, University of York, York, YO10 5DD UK; 2 Solent NHS Trust, Portsmouth, PO3 6AD UK; 3 Oxford Health NHS Foundation Trust, Oxford, OX3 7JX UK; 4 Lancashire Care NHS Foundation Trust, Preston, PR2 8DW UK; 5 Kent and Medway NHS and Social Care Partnership Trust, Maidstone, ME16 9PH UK; 6 Northumberland, Tyne and Wear NHS Foundation Trust, Newcastle, NE3 3XT UK; 7 Tees, Esk and Wear Valleys NHS Foundation Trust, Harrogate, HG1 2PW UK; 8 Division of Psychiatry, University College London, London, WC1E 6BT UK; 9 Leeds York NHS Foundation Trust, Leeds, LS15 8ZB UK; 10 Cambridgeshire and Peterborough NHS Foundation Trust, Cambridge, CB21 5EF UK; 11 Southern Health NHS Foundation Trust, Southampton, SO30 3JB UK; 12 2gether NHS Foundation Trust, Gloucester, GL1 1LY UK

**Keywords:** Randomised controlled trial, Recruitment, Severe mental ill health, Smoking cessation, Psychosis, Bipolar

## Abstract

**Background:**

Randomised controlled trials (RCT) can struggle to recruit to target on time. This is especially the case with hard to reach populations such as those with severe mental ill health. The SCIMITAR+ trial, a trial of a bespoke smoking cessation intervention for people with severe mental ill health achieved their recruitment ahead of time and target. This article reports strategies that helped us to achieve this with the aim of aiding others recruiting from similar populations.

**Methods:**

SCIMITAR+ is a multi-centre pragmatic two-arm parallel-group RCT, which aimed to recruit 400 participants with severe mental ill health who smoke and would like to cut down or quit. The study recruited primarily in secondary care through community mental health teams and psychiatrists with a smaller number of participants recruited through primary care. Recruitment opened in October 2015 and closed in December 2016, by which point 526 participants had been recruited. We gathered information from recruiting sites on strategies which led to the successful recruitment in SCIMITAR+ and in this article present our approach to trial management along with the strategies employed by the recruiting sites.

**Results:**

Alongside having a dedicated trial manager and trial management team, we identified three main themes that led to successful recruitment. These were: clinicians with a positive attitude to research; researchers and clinicians working together; and the use of NHS targets. The overriding theme was the importance of relationships between both the researchers and the recruiting clinicians and the recruiting clinicians and the participants.

**Conclusions:**

This study makes a significant contribution to the limited evidence base of real-world cases of successful recruitment to RCTs and offers practical guidance to those planning and conducting trials. Building positive relationships between clinicians, researchers and participants is crucial to successful recruitment.

## Background

One of the most important challenges faced when conducting a randomised controlled trial (RCT) is recruiting the desired number of participants in the designated time frame [1–3]. An analysis of trials funded by two large UK agencies indicated that 45% of trials failed to meet their recruitment target and 46% had to extend the study duration in order to meet the targets [4]. Under-recruitment can lead to an underpowered study and problems estimating the effectiveness of the intervention or treatment being tested [5]. Some studies might even be abandoned or closed prematurely. In cases where studies seek an extension to facilitate recruitment, there is usually an increase in trial costs and a delay in obtaining results.

Systematic reviews [6–8] have identified barriers to recruitment from the perspectives of both recruiters and participants. Briel et al. [8] identified 28 reasons for recruitment failure, categorised into four themes: funding; design; recruiter; and participant. Common recruitment failure reasons were: overestimation of eligible participants; recruiter and participant opinions on trial intervention effectiveness; and burden of trial involvement for recruiters and participants. Ross et al. [9] and Brintnall-Karabelas et al. [7] also found similar explanations for recruitment failure.

Despite the impact under-recruitment can have on trials, and evidence highlighting specific barriers to trial recruitment, there is a lack of information about how trials that have recruited to time and target have achieved this. The introduction of the CONSORT statement, to improve reporting of clinical trials [10], has been recognised as leading to an improvement in the reporting of RCTs in a psychiatric population [11], but it does not stipulate the reporting of recruitment strategies, making sharing of best practice limited. Systematic reviews [12–14] have identified some trials that evaluated different recruitment methods, although these were often small, under-powered or used quasi-randomised or hypothetical designs which limit the generalisability to ‘real-world’ RCTs.

One systematic review by Belisario et al. [15] investigated methods for recruitment to smoking cessation studies. High levels of personal contact resulted in better recruitment rates, although only one study (personalised phone call vs generic letter) demonstrated a statistically significant difference (relative risk [RR] = 40.73, 95% confidence interval [CI] = 2.53–654.74 [16]). Using a combination of recruitment strategies concurrently, for example using telephone and text messaging, was consistently found to increase recruitment rates (RR = 3.38, 95% CI = 1.26–9.08 [17], RR = 29.07, 95% CI = 1.74–485.70 [17]).

The SCIMITAR+ trial follows on from the SCIMITAR pilot trial with the design of the intervention being reported elsewhere [18, 19]. The SCIMITAR+ trial aimed to recruit 400 people with severe mental ill health [20] (SMI). This population has traditionally been hard to recruit [21], but SCIMITAR+ met its target recruitment figures ahead of schedule. We therefore report in this article the strategies and approach to trial management used to support recruitment to SCIMITAR+, with the purpose of extending the evidence base for effective trial recruitment and aiding others recruiting to trials in similar populations.

## Methods

Full details of the protocol for the SCIMITAR+ study have been reported elsewhere [20]. Recruitment began on 1 October 2015 with a stepped increase in the target number of participants to be recruited per month from ten in October 2015 to 25 per month by March 2016. Recruitment was scheduled to end on 31 March 2017; however, the target recruitment of 400 participants was reached in mid-October 2016, almost five months ahead of schedule. The inclusion criteria for the SCIMITAR+ Trial are shown in Table 1.

**Table 1:** Inclusion and exclusion criteria

Inclusion criteria	Exclusion criteria
• Age over 18 years • Documented diagnosis of SMI (schizophrenia, delusional/psychotic illness or bipolar disorder) • Current smoker (at least 5 per day) • English speaker	• Pregnant or breastfeeding • Have drug or alcohol abuse as a current primary diagnosis • Lacks mental capacity to consent

Participants who wanted to cut down or quit smoking were randomised to receive either a bespoke smoking cessation intervention tailored to the needs of people with SMI or usual care available in their area. Ethical approval was sought and granted by Leeds East Research Ethics committee (15/YH/0051). Informed consent was sought from all participants before recruitment.

### Recruitment methods

Five main recruitment strategies were employed. We recruited via: (1) GP surgeries; (2) community mental health teams or psychiatrists; (3) service user groups; (4) poster advertisements; and (5) a lifestyle survey. Irrespective of the strategy used, participants’ suitability to take part in the trial had to be established by a clinician.

## Results

**Fig. 1:**
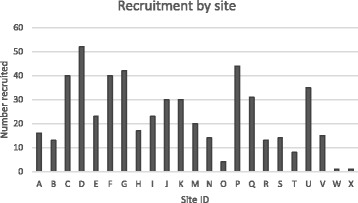
Recruitment by site in the SCIMITAR+ trial

The SCIMITAR+ trial recruited 526 participants between October 2015 and December 2016 across 22 sites in England. Sites recruited varying numbers of participants as shown in Fig. 1.

We present below the chronological stages of the research and how we maximised opportunities to recruit at each stage.

### Selecting the research question

The SCIMITAR pilot trial was undertaken in response to a commissioned call from the National Institute of Health Research (HTA reference 07/41/05), indicating the importance of the research question in a UK context. In recent years the need to improve the physical health of those with mental illness has received national attention in the UK [22]. Implementation of Commissioning for Quality and Innovation (CQUIN) targets has seen mental health trusts respond by improving physical healthcare and working towards national physical health targets. In addition, National Institute for Health and Care Excellence guidance [23] recommended in 2013 that NHS sites become entirely smoke-free by 2018 and that service users who smoked should be offered help to quit. This cemented the importance of smoking cessation interventions for people with mental health problems and led to the perception that the research question was of importance for both the research community and healthcare staff.

#### Designing the recruitment process

SCIMITAR+ was a collaboration between the Mental Health and Addictions Research Group and York Trials Unit, both situated in the Department of Health Sciences at the University of York. This collaboration combined researchers who specialise in mental health studies with researchers who specialise in trials. One of the key factors observed by Farrell et al. [24] in relation to managing clinical trials effectively was the employment of a dedicated trial manager. Thus, a dedicated trial manager and trial coordinators with responsibilities for specific aspects of the trial were employed to ensure the smooth running of recruitment and allocation to the intervention.

To recruit well from mental health teams, it is important to establish a close working relationship with clinicians. Cooperating with clinicians and supporting them to feel confident and involved with the SCIMITAR+ study was key in the recruitment strategy for this trial. Identifying and building a network of professionals with enthusiasm for research played a crucial role. Multiple methods were used to support this, including: establishing research champions within clinical teams; building relationships with individual professionals in potential recruitment areas; and initiating conversations between clinicians and researchers about the trial. Displaying and developing enthusiasm for SCIMITAR+ and the potential positive outcomes that could be gained through engagement in the trial was an important part of these discussions. Such actions fostered good relationships with clinicians and enabled access to potential participants.

In many sites, researchers were embedded into mental health teams who focused on engaging that team and promoting study participation. This ranged from a full-time commitment through to spending time on wards and units interacting with staff and patients informally (achieved with the service support costs – from the local Comprehensive Research Network [CRN]). Initially, this allowed the researchers to identify how recruitment would function best within that team, e.g. where to place advertisements so that they were most likely to be read. As the study continued, the presence of the researcher served as a regular reminder of SCIMITAR+ and meant that the clinical team had a recognised contact for the study to whom they could direct questions and any participant referrals. Informal conversations aided with networking, and gave the potential for discussions about SCIMITAR+, to identify team members who were interested in supporting the research.

A wholehearted approach to recruitment seemed to be most effective in two areas. First, where the researcher could speak directly with potential participants, e.g. when potential participants attended a clinic for a routine long-acting injection, a motivated clinician actively identifying potential participants and discussing SCIMITAR+ with them, particularly where the participant had a trusting relationship with the clinician, had a positive effect on recruitment. People recruited in this way appeared more engaged and genuinely interested in the trial and were more likely to seek further information. Second, it was particularly useful when the person promoting the trial was the Principal Investigator or was in a position to influence other clinicians to encourage engagement. Most notably, it was identified that interaction with researchers could provide both individual clinicians and teams with evidence that they were meeting their professional responsibilities in relation to research. These include the responsibilities outlined within the NHS constitution [25], i.e. supporting access to pertinent research projects, developing evidence-based practice and enabling patients to make informed decisions about their care and treatment.

Researchers also supported clinicians with recruitment during outpatient clinics, such as clozapine and depot clinics. Initially, study staff would remind the clinicians of the details and eligibility criteria of the study so that they felt confident in making referrals. Researchers would then either sit in the clinic room to speak directly to patients or wait in a separate room for patients to be referred to them after their appointment. Primary care services were also recognised as potential settings for recruitment and would-be avenues worthy of consideration in future trials as necessary.

### Engaging with sites

The trial management team (trial manager and trial coordinators) worked to ensure that all procedures were as straightforward as possible, particularly in relation to study recruitment. The burden on recruiters was minimised by providing recruitment packs containing all the necessary materials required for recruitment and ensuring that the trial materials were attractive, clear and easy to understand to both recruitment sites and potential participants. Frequent meetings were held between the study’s trial management team and recruiting sites, with the aim of sharing effective strategies, answering queries and supporting sites that were experiencing difficulties with recruitment. The trial management team aimed to establish a supportive culture by responding to queries promptly and taking time to listen to concerns.

The opening of sites was phased with a small number of sites opening initially so that any problems with recruitment procedures could be discussed and rectified early in the process before additional sites joined. This also allowed a projection of the number of sites needed to meet the recruitment targets by calculating the number of participants the early sites were able to recruit in the first three months.

Several of the mental health trusts involved in SCIMITAR+ had large-scale ‘smoke-free’ programmes ongoing during the trial. Research staff collaborated with the smoke-free team, by either inviting them to join the site’s trial management group or by attending meetings with physical health nurses and smoking cessation advisors. Involvement in these meetings enabled SCIMITAR+ researchers to remind staff to discuss the trial with eligible service users. The weekly staff bulletin was also used to provide updates on both the smoke-free campaign and how to refer patients to SCIMITAR+.

### Continuing engagement

Team meetings provided useful opportunities to build relationships with clinicians and ensure continued engagement with the study. The majority of sites gave presentations to mental health teams at the beginning of the study, summarising the key aims, benefits and eligibility criteria of SCIMITAR+. This ensured that clinical staff understood the study and how they could help with recruitment. In many cases, researchers continued to attend team meetings to give updates and promote engagement with the study, thus establishing SCIMITAR+ as an important part of the team’s work. Meetings also provided a forum for addressing the barriers, questions and concerns identified by clinicians, and were a chance for researchers to offer practical support.

In addition, researchers used the relationships they had established with clinicians to work with them on a one-to-one level. Primarily, this involved screening caseloads for eligible participants. Researchers could then arrange with clinicians to attend their next meeting with the potential participant (with permission) in order to discuss the study in person. Clinicians reported that they felt more supported with this approach, as the researcher is better equipped to speak about the details of the trial and answer any questions the participant may have. This is particularly useful in services in which several research studies are running concurrently, as clinicians may find it difficult to talk confidently about each. Speaking with clinicians individually also allowed researchers to highlight the benefits for clinicians of assisting with research, e.g. building their professional development portfolios.

Developing links with local health screening clinics at an early stage in recruitment was also beneficial. Potential trial participants were identified by clinic staff and the option of referral to SCIMITAR+ was discussed during smoking cessation conversations. The CQUIN targets further encouraged clinicians to ask every patient whether they smoked during routine care appointments. This prompted a conversation about smoking cessation and the opportunity to participate in SCIMITAR+, thus increasing recruitment to the trial with minimal impact on the routine work of the clinicians while maintaining a mutually supportive relationship with the research team. We had also hoped that the UK’s Stoptober initiative would generate increased interest in the trial around this time, but did not find this. Future trials could consider how best to take advantage of relevant promotions such as this.

One particularly successful partnership involved a new unit dedicated to integrating the physical and mental healthcare of people with psychosis. The service had a target to support smokers to quit so the unit was able to promote SCIMITAR+ to patients which in turn helped the service to meet their smoking cessation target. Once the benefits of collaboration with SCIMITAR+ were realised, the unit’s managers encouraged other managers to raise awareness of SCIMITAR+ in their departments, thus expanding the recruitment drive within the NHS trust.

## Discussion

This article explores strategies for successful recruitment in the context of a large, multi-centre RCT (SCIMITAR+). Three key factors were identified to be important in increasing recruitment to both SCIMITAR+ and for future RCTs: clinicians’ attitudes; inter-professional working; and utilising NHS targets. Within the three themes, building positive relationships between research and clinical staff was consistently reported as crucial for effective and efficient recruitment.

As noted by Patterson et al. [2], patient referral rates to research studies may be dictated by clinicians’ attitudes to research and this was observed within the SCIMITAR+ trial. Building relationships between the SCIMITAR+ research team and clinicians helped to increase enthusiasm for the study and where this enthusiasm subsequently transferred from the clinical team to potential participants, recruitment rate increased, as can be seen in Fig. 1 where sites with greater levels of enthusiasm tended to recruit better. While recruitment to RCTs can benefit from targeted involvement of clinicians with an interest in the study topic, further work through the life course of a trial is necessary to build and maintain relationships that sustain interest and enthusiasm among the research staff. Continuing a presence of research staff within clinical teams has been found to be crucial in helping to build and maintain necessary relationships [26]. Where clinicians are already invested in the research project, it is prudent to utilise this presence and enthusiasm to facilitate study presence and to engage members of the wider clinical team. Development of these relationships would assist in enhancing interest and investment in research activity and may therefore result in increased recruitment activity in both current and future projects. It is important to note that the SCIMITAR+ trial is a non-commercial trial conducted within the NHS. There were therefore no financial incentives for clinicians to recruit which could lead to a conflict of interest. In studies where such a conflict could arise, it would be essential to implement strategies to mitigate against such conflicts.

The impact of research responsibilities on clinical teams continues to be a barrier to recruitment, with many clinicians reporting that clinical workloads mean they struggle to accommodate research activity [6, 27]. Our research has identified that development and maintenance of relationships between the research and clinical teams helps to facilitate support for recruitment activity (e.g. screening caseloads, addressing queries, identifying recruitment locations). This corresponds with previous research in mental health studies, which identified that establishing good working relationships before commencing recruitment to studies and then maintaining this over time is important to facilitate successful study conduct [26].

Where relationships were developed and support provided during SCIMITAR+, recruitment rates were comparatively better compared to sites where support was not so forthcoming. The implication of increased recruitment rates is that involved NHS sites may improve performance in relation to predefined NHS targets, thus increasing future funding levels, which may increase support available for future research studies. Future RCTs should therefore consider from the outset how best to utilise dedicated research staff to support clinical teams with recruitment activity, to ensure a targeted approach appropriate to the setting. In addition, previous research studies exploring barriers and facilitators to study recruitment have identified that relevance of the study to clinicians and availability of resources to undertake research activity are critical in facilitating recruitment in clinical settings [6, 27, 28]. Development of positive, professional relationships is undoubtedly likely to help to minimise these barriers and in turn increase recruitment of participants.

The management of a trial can also have an important effect on the recruitment rate [29]. In SCIMITAR+, we had a dedicated trial manager and trial management team who ensured that trial procedures were as simple as possible and queries dealt with promptly, both of which have previously been found to be effective in aiding recruitment [28]. As recommended by Farrell [24], a management plan was in place which was constantly reviewed and revised as the trial progressed. Alongside this, we aimed to build enthusiasm and a supportive culture both in the trial management team and among researchers working on the trial. It is recommended that this approach to trial management is adopted for future RCTs.

## Conclusions

Building positive relationships from the outset of a trial is crucial to ensure that recruitment is successful. This study makes a valuable contribution to the limited evidence base of real-world examples of successful recruitment methods for RCTs and offers practical guidance to researchers planning and conducting RCTs. This evidence, and continued sharing of effective techniques, will help to ensure that all trials have the best chance of recruiting to time and target.

Future strategies that include electronic recruitment methods that utilise patient electronic health records (e.g. Join Dementia Register [JDR]; Consent for Contact [SLaM C4C]; and Clinical Record Interactive Search [CRIS]) may also prove useful [30].

## Abbreviations

CMHT: Community mental health team
CQUIN: Commissioning for quality and innovation
CRN: Comprehensive research network
RCT: Randomised controlled trial
SMI: Severe mental ill health
